# Impact of axillary surgery on survival in de novo metastatic breast cancer with primary tumor resection

**DOI:** 10.1007/s10549-026-07998-2

**Published:** 2026-05-24

**Authors:** Mahtab Vasigh, Karen Ruth, Richard J. Bleicher, Walker Lyons, Allison A. Aggon, Austin D. Williams

**Affiliations:** 1 Division of Breast Surgery, Department of Surgery, Fox Chase Cancer Center, 333 Cottman Avenue, Philadelphia, PA 19111 USA; 2 Biostatistics Facility, Fox Chase Cancer Center, Philadelphia, PA USA

**Keywords:** De novo metastatic breast cancer, Axillary surgery, Overall survival, Sentinel lymph node biopsy, Axillary lymph node dissection

## Abstract

**Purpose:**

The role of axillary surgery in patients with de novo metastatic breast cancer (dnMBC) undergoing primary site surgery is unclear. We evaluated patterns of axillary management and associations with overall survival (OS) in this population.

**Methods:**

We used the National Cancer Database (2012-2020) to identify patients with dnMBC. Patients were grouped by receipt of breast surgery and axillary surgery (none, sentinel lymph node biopsy [SLNB] or axillary lymph node dissection [ALND]). Multivariable Cox proportional hazards models assessed factors associated with axillary surgery and OS.

**Results:**

Among 39,946 eligible patients, 7,808 (19.5%) underwent surgery of the primary site, of whom 79.8% underwent axillary surgery (79.5% ALND, 20.4% SLNB). Compared with no axillary surgery, those who underwent axillary surgery had more favorable clinicodemographic characteristics (younger, fewer comorbidities, lower T stage, bone-only metastases). In patients with clinically node-negative disease, 41.6% had pathologically negative nodes, with higher rates among those with HR+/HER- disease and those who did not have systemic therapy prior to surgery. On multivariable analysis, axillary surgery was independently associated with improved OS (HR 0.70; 95% CI 0.63–0.78; p<0.001).

**Conclusion:**

Among patients with dnMBC undergoing primary site surgery, axillary surgery was associated with improved survival in this retrospective analysis, though the effect is likely influenced by selection bias. Until prospective data define which subgroups may benefit, axillary surgery should be considered selectively, with SLNB preferred over ALND for patients without palpable adenopathy to minimize morbidity. Multidisciplinary evaluation remains essential to tailor surgical decisions that balance survival, function, and quality of life.

**Supplementary Information:**

The online version contains supplementary material available at 10.1007/s10549-026-07998-2.

## Introduction

Approximately 5–10% of patients with breast cancer present with *de* novo metastatic disease (dnMBC) [1]. The 5-year survival rate for dnMBC is about 27%, compared to 99% and 86% for local and regional disease, respectively [2]. Despite recent advances in systemic therapy that have extended overall survival (OS), dnMBC remains incurable, with treatment primarily focused on prolonging life, symptom control, and supportive care [3]. The role of locoregional treatment, particularly surgery, in this setting remains controversial.

Multiple retrospective studies have suggested that resection of the primary tumor resection is associated with improved survival in dnMBC, particularly among patients with favorable hormone receptor status, limited metastatic burden, good systemic therapy response, and negative surgical margins [4–10]. However, these findings have not been consistently produced in randomized controlled trials, most of which have failed to demonstrate a survival benefit from locoregional therapy [11–14]. This discordance between retrospective and prospective data strongly suggests that observed survival advantages in observational studies are driven, at least in part, by selection bias and confounding by indication rather than a true causcal effect of surgery.

When primary site surgery is performed in dnMBC, axillary surgery is often included, based on paradigms established in early-stage disease. Randomized trials of surgery in dnMBC mandated sentinel lymphadenectomy (SLNB) for clinically node-negative (cN–) patients and axillary lymph node dissection (ALND) for clinically node-positive (cN+) patients or those with positive SLNB findings. Notably, even the earliest randomized clinical trials in non-metastatic breast cancer demonstrated that axillary surgery does not confer a survival benefit, raising fundamental questions about its value in de novo metastatic disease, where overall survival is driven primarily by systemic therapy and nodal status rarely alters treatment decisions [14]. 

A 2020 meta-analysis of 16 studies conducted between 1989 and 2015 found no survival advantage associated with axillary surgery in dnMBC [15]. However, evolving systemic therapy and surgical selection may limit the relevance of older data. We therefore evaluated contemporary practice patterns, pathologic findings, and survival outcomes associated with axillary surgery in dnMBC patients undergoing primary site resection.

## Methods

We used the 2020 Participant User File of the National Cancer Database (NCDB) to identify patients diagnosed with de novo metastatic breast cancer (dnMBC; clinical and/or pathological M1) between 2012 (revised score of regional LN surgery variable was first available) and 2019 (last year of mortality follow-up). Since these data are de-identified, this study was deemed exempt from Institutional Review Board approval. Patients were excluded if they had missing data on primary site or axillary surgery, unknown lymph node surgery status, a history of another malignancy, no surgery of the primary site, no systemic treatment, or surgery more than 1 year after diagnosis. The NCDB captures metastatic site at diagnosis (e.g., bone, liver, lung, brain), but does not provide information on the number, volume, or extent of metastatic lesions, nor on histologic confirmation of metastatic disease. Cohort selection, exclusions, and analytic populations are detailed in Supplementary Fig. 1.

An initial exploratory analysis compared clinicopathologic characteristics between patients who did and did not undergo primary site surgery. We examined trends by year of diagnosis in the proportion of patients having primary site surgery, axillary surgery, and the axillary surgery approach, using Cochran-Armitage trend tests. For the primary analyses comparing by axillary surgery status, we included only patients who had primary site surgery and stratified them by axillary surgical approach: none, sentinel lymph node biopsy (SLNB) alone, or axillary lymph node dissection (ALND) with or without SLNB (performed concurrently or subsequently).We considered axillary surgery status (yes/no), and also within those who had axillary surgery (SLNB vs. ALND ± SLNB). In patients who had axillary surgery, we evaluated characteristics by pathologic nodal status (positive nodes found vs. not); those with missing pN or LN counts were excluded.

For these comparisons, characteristics were summarized as frequencies and percentages for categorical variables and means ± standard deviation (SD) for continuous variables. Comparisons used Chi-square or Fisher’s exact tests for categorical variables and t-tests for continuous variables.

To examine the independence of the characteristics associated with positive axillary nodes, we used multivariable logistic regression in axillary surgery patients with complete cT, cN and phenotype data who were age 40 + at diagnosis, as the younger patients have unknown facility type per NCDB PHI guidelines. In a second logistic model, we included only those who were cN0.

The association between axillary surgery and overall survival (OS) was examined using a landmark analysis defined as the interval from 12 months after diagnosis to death, with survivors censored at last follow-up. A 12-month landmark was selected to align with the design of prior randomized trials in de novo metastatic breast cancer, in which patients were required to demonstrate no disease progression for a defined period prior to enrollment. This approach helps reduce immortal time bias by excluding patients with rapidly progressive disease who are unlikely to benefit from surgery and allows for a more clinically relevant comparison among patients who are potential candidates for locoregional intervention. Unadjusted OS was compared between groups using Kaplan–Meier methods. Propensity score weighting was used to adjust for covariate imbalances; stabilized inverse probability weights were calculated, and covariate balance was assessed after weighting. All covariates were balanced with the exception of treatment order (surgery first or systemic first), and the imbalance was considered acceptable (Supplementary Table 1).To account for potential clustering of outcomes by treatment facility, models were adjusted using robust standard errors at the facility level.

Hormone receptor–positive (HR+) status was defined as ER + and/or PR+; HER2 + as HER2-positive regardless of HR status; and triple-negative as ER−/PR−/HER2−. Patients with missing data for variables for a given analysis were excluded from that analysis. Analyses were performed using SAS software, version 9.4 (Cary, NC, USA).

## Results

### Primary site surgery trends and patient characteristics

**Fig. 1:**
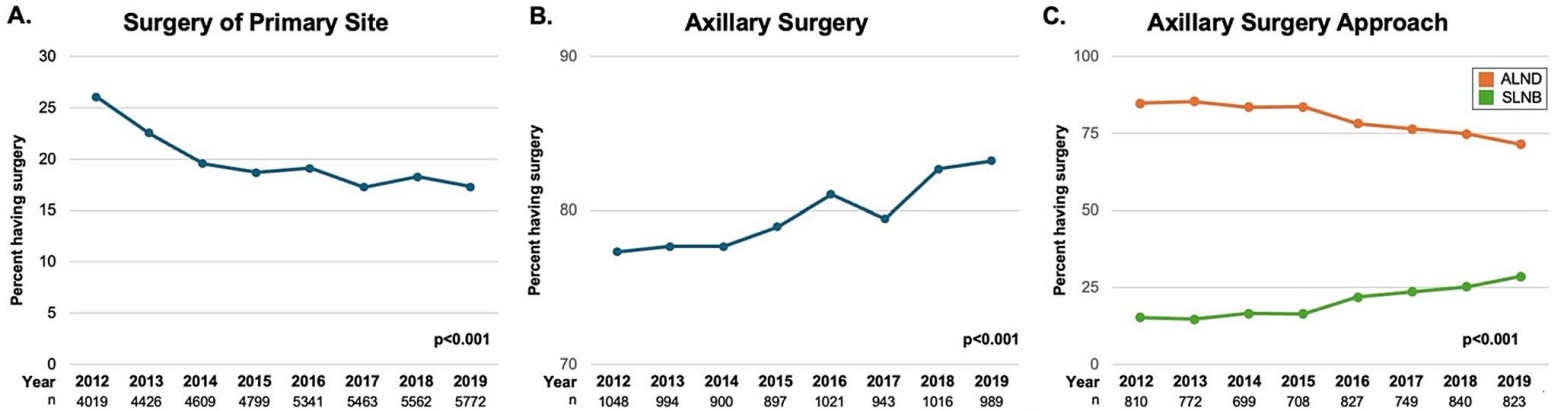
Surgery trends over time among patients with de novo metastatic breast cancer: **A**: Primary site surgery, **B**: Axillary surgery in patients who had primary site surgery, **C**: Type of axillary surgery. ALND: Axillary lymph node dissection, SLNB: Sentinel lymph node dissection

From 2012 to 2020, 39,946 patients with dnMBC were identified; 7,808 (20%) underwent primary site surgery (Table 1). The rate of surgery declined over this period from 26% to 17% (*p* < 0.001, Fig. 1A). Patients undergoing surgery were younger, more often White, had fewer comorbidities, were more likely to be privately insured, and were less frequently treated at Academic/Research programs. They were also more likely to have bone-only metastases, higher tumor stage, ductal histology, and HER2 + or triple-negative subtypes. Nodal stage distribution in the surgical cohort showed higher proportions of both cN0 and ≥cN2 disease. Mastectomy was the predominant surgical approach (71%). Median time from diagnosis to surgery was similar between groups (SLNB: 64 days vs. ALND: 57 days; Wilcoxon *p* = 0.07).

**Table 1:** Clinicopathologic characteristics of metastatic breast cancer patients stratified by whether they underwent surgery of the primary tumor site

		Overall	Surgery of the primary site		*p*
			No	Yes	
**n**		39,946	32,138	7,808	
**Age** (years)		58.0 ± 12.0	58.7 ± 11.8	54.8 ± 12.5	< 0.001
**Sex**					
	Male	605 (1.5)	443 (1.4)	162 (2.1)	< 0.001
	Female	39,341 (98.5)	31,695 (98.6)	7646 (97.9)	
**Race**					
	White	29,844 (74.7)	23,862 (74.2)	5982 (76.6)	< 0.001
	Black	7540 (18.9)	6239 (19.4)	1301 (16.7)	
	Asian	1489 (3.7)	1165 (3.6)	324 (4.1)	
	Other/Unknown	1073 (2.7)	872 (2.7)	201 (2.6)	
**Charlson/Deyo Score**					
	0	32,977 (82.6)	26,327 (81.9)	6650 (85.2)	< 0.001
	1	4857 (12.2)	3954 (12.3)	903 (11.6)	
	2+	2112 (5.3)	1857 (5.8)	255 (3.3)	
**Insurance Status**					
	Not Insured	2158 (5.4)	1841 (5.7)	317 (4.1)	< 0.001
	Private Insurance	17,933 (44.9)	13,666 (42.5)	4267 (54.6)	
	Medicaid	6137 (15.4)	5065 (15.8)	1072 (13.7)	
	Medicare	12,764 (32.0)	10,807 (33.6)	1957 (25.1)	
	Other Government	394 (1.0)	288 (0.9)	106 (1.4)	
	Unknown	560 (1.4)	471 (1.5)	89 (1.1)	
**No High School Degree**					
	>=17.6%	8265 (20.7)	6726 (20.9)	1539 (19.7)	0.04
	10.9% − 17.5%	9470 (23.7)	7644 (23.8)	1826 (23.4)	
	6.3% − 10.8%	9640 (24.1)	7750 (24.1)	1890 (24.2)	
	< 6.3%	7196 (18.0)	5749 (17.9)	1447 (18.5)	
	Unknown	5375 (13.5)	4269 (13.3)	1106 (14.2)	
**Median Income**					
	< $40,227	7081 (17.7)	5765 (17.9)	1316 (16.9)	0.06
	$40,227 - $50,353	7621 (19.1)	6142 (19.1)	1479 (18.9)	
	$50,354 - $63,332	8050 (20.2)	6430 (20.0)	1620 (20.7)	
	>=$63,333	11,761 (29.4)	9481 (29.5)	2280 (29.2)	
	Unknown	5433 (13.6)	4320 (13.4)	1113 (14.3)	
**Institution Type**					
	Community Cancer Center	2666 (6.7)	2093 (6.5)	573 (7.3)	< 0.001
	Comprehensive Community Cancer Program	13,574 (34.0)	10,722 (33.4)	2852 (36.5)	
	Academic/Research Program	13,380 (33.5)	11,474 (35.7)	1906 (24.4)	
	Integrated Network Cancer Program	6821 (17.1)	5435 (16.9)	1386 (17.8)	
**Histology**					
	Ductal	32,664 (81.8)	25,803 (80.3)	6861 (87.9)	< 0.001
	Lobular	3913 (9.8)	3358 (10.4)	555 (7.1)	
	Mixed/Other	3369 (8.4)	2977 (9.3)	392 (5.0)	
**Tumor Grade**					
	Low	1533 (3.8)	1255 (3.9)	278 (3.6)	< 0.001
	Intermediate	9813 (24.6)	7687 (23.9)	2126 (27.2)	
	High	11,766 (29.5)	8350 (26.0)	3416 (43.7)	
	Unknown	16,834 (42.1)	14,846 (46.2)	1988 (25.5)	
**Lymphovascular Invasion**					
	Absent	8984 (22.5)	6408 (19.9)	2576 (33.0)	< 0.001
	Present	5573 (14.0)	2528 (7.9)	3045 (39.0)	
	Unknown	25,389 (63.6)	23,202 (72.2)	2187 (28.0)	
**Clinical Tumor Stage**					
	cT0	782 (2.0)	766 (2.4)	16 (0.2)	< 0.001
	cT1	4159 (10.4)	3294 (10.2)	865 (11.1)	
	cT2	11,328 (28.4)	8736 (27.2)	2592 (33.2)	
	cT3	6236 (15.6)	4874 (15.2)	1362 (17.4)	
	cT4	12,484 (31.3)	9964 (31.0)	2520 (32.3)	
	Unknown	4957 (12.4)	4504 (14.0)	453 (5.8)	
**Clinical Nodal Stage**					
	cN0	7864 (19.7)	6177 (19.2)	1687 (21.6)	< 0.001
	cN1	17,697 (44.3)	14,383 (44.8)	3314 (42.4)	
	cN2	4455 (11.2)	3470 (10.8)	985 (12.6)	
	cN3	6060 (15.2)	4696 (14.6)	1364 (17.5)	
	Unknown	3870 (9.7)	3412 (10.6)	458 (5.9)	
**Tumor Subtype**					
	HR+/HER2-	21,792 (54.6)	17,905 (55.7)	3887 (49.8)	< 0.001
	HER2+	10,164 (25.4)	7761 (24.1)	2403 (30.8)	
	TNBC	4919 (12.3)	3681 (11.5)	1238 (15.9)	
	Unknown	3071 (7.7)	2791 (8.7)	280 (3.6)	
**Site of metastasis**					
	Bone only	15,089 (37.8)	11,771 (36.6)	3318 (42.5)	< 0.001
	Visceral or multiple sites	21,693 (54.3)	18,477 (57.5)	3216 (41.2)	
	Unknown	3164 (7.9)	1890 (5.9)	1274 (16.3)	
**Breast Surgery**					
	Partial Mastectomy	2250 (5.6)	-	2250 (28.8)	-
	Mastectomy	5558 (13.9)	-	5558 (71.2)	
	None	32,138 (80.5)	32,138 (100.0)	-	

### Axillary surgery utilization

Among patients with primary site surgery, 6,228 (80%) had axillary surgery (Table 2), most commonly ALND±SLNB (80%, 4,955/6,228). In 78% of ALND cases, no prior SLNB was performed. Over time, axillary surgery rates increased from 77% to 83% (*p* < 0.001, Fig. 1B), while ALND declined from 84% to 71% (*p* < 0.001, Fig. 1C).

**Table 2:** Clinicopathologic characteristics of metastatic breast cancer patients undergoing primary cancer surgery stratified by type of axillary surgery performed

		Overall	Axillary Surgery				*p* ^1^	*p* ^2^
			No	Yes				
				Overall	SLNB	ALND±SLNB		
**n**		7,808	1,580	6,228	1,273	4,955		
**Age (years)**		54.8 ± 12.5	57.2 ± 12.1	54.2 ± 12.5	53.1 ± 12.6	54.4 ± 12.5	< 0.001	0.001
**Sex**								
	Male	162 (2.1)	37 (2.3)	125 (2.0)	16 (1.3)	109 (2.2)	0.40	0.03
	Female	7646 (97.9)	1543 (97.7)	6103 (98.0)	1257 (98.7)	4846 (97.8)		
**Race**								
	White	5982 (76.6)	1228 (77.7)	4754 (76.3)	1027 (80.7)	3727 (75.2)	0.16	< 0.001
	Black	1301 (16.7)	259 (16.4)	1042 (16.7)	162 (12.7)	880 (17.8)		
	Asian	324 (4.1)	50 (3.2)	274 (4.4)	48 (3.8)	226 (4.6)		
	Other/Unknown	201 (2.6)	43 (2.7)	158 (2.5)	36 (2.8)	122 (2.5)		
**Charlson/Deyo Score**								
	0	6650 (85.2)	1338 (84.7)	5312 (85.3)	1103 (86.6)	4209 (84.9)	0.41	0.31
	1	903 (11.6)	182 (11.5)	721 (11.6)	133 (10.4)	588 (11.9)		
	2+	255 (3.3)	60 (3.8)	195 (3.1)	37 (2.9)	158 (3.2)		
**Insurance Status**								
	Not Insured	317 (4.1)	74 (4.7)	243 (3.9)	40 (3.1)	203 (4.1)	0.008	< 0.001
	Private Insurance	4267 (54.6)	804 (50.9)	3463 (55.6)	796 (62.5)	2667 (53.8)		
	Medicaid	1072 (13.7)	215 (13.6)	857 (13.8)	147 (11.5)	710 (14.3)		
	Medicare	1957 (25.1)	446 (28.2)	1511 (24.3)	255 (20.0)	1256 (25.3)		
	Other Government	106 (1.4)	20 (1.3)	86 (1.4)	18 (1.4)	68 (1.4)		
	Unknown	89 (1.1)	21 (1.3)	68 (1.1)	17 (1.3)	51 (1.0)		
**No High School Degree**								
	>=17.6%	1539 (19.7)	295 (18.7)	1244 (20.0)	225 (17.7)	1019 (20.6)	0.06	< 0.001
	10.9% − 17.5%	1826 (23.4)	362 (22.9)	1464 (23.5)	248 (19.5)	1216 (24.5)		
	6.3% − 10.8%	1890 (24.2)	415 (26.3)	1475 (23.7)	326 (25.6)	1149 (23.2)		
	< 6.3%	1447 (18.5)	267 (16.9)	1180 (18.9)	272 (21.4)	908 (18.3)		
	Unknown	1106 (14.2)	241 (15.3)	865 (13.9)	202 (15.9)	663 (13.4)		
**Median Income**								
	< $40,227	1316 (16.9)	252 (15.9)	1064 (17.1)	173 (13.6)	891 (18.0)	0.19	< 0.001
	$40,227 - $50,353	1479 (18.9)	317 (20.1)	1162 (18.7)	218 (17.1)	944 (19.1)		
	$50,354 - $63,332	1620 (20.7)	303 (19.2)	1317 (21.1)	260 (20.4)	1057 (21.3)		
	>=$63,333	2280 (29.2)	467 (29.6)	1813 (29.1)	417 (32.8)	1396 (28.2)		
	Unknown	1113 (14.3)	241 (15.3)	872 (14.0)	205 (16.1)	667 (13.5)		
**Institution Type**								
	Community Cancer Center	573 (7.3)	144 (9.1)	429 (6.9)	69 (5.4)	360 (7.3)	< 0.001	0.02
	Comprehensive Community Cancer Program	2852 (36.5)	648 (41.0)	2204 (35.4)	442 (34.7)	1762 (35.6)		
	Academic/Research Program	1906 (24.4)	339 (21.5)	1567 (25.2)	300 (23.6)	1267 (25.6)		
	Integrated Network Cancer Program	1386 (17.8)	295 (18.7)	1091 (17.5)	248 (19.5)	843 (17.0)		
	Unknown	1091 (14.0)	154 (9.7)	937 (15.0)	214 (16.8)	723 (14.6)		
**Histology**								
	Ductal	6861 (87.9)	1356 (85.8)	5505 (88.4)	1147 (90.1)	4358 (88.0)	< 0.001	0.002
	Lobular	555 (7.1)	102 (6.5)	453 (7.3)	94 (7.4)	359 (7.2)		
	Mixed/Other	392 (5.0)	122 (7.7)	270 (4.3)	32 (2.5)	238 (4.8)		
**Tumor Grade**								
	Low	278 (3.6)	70 (4.4)	208 (3.3)	49 (3.8)	159 (3.2)	0.008	< 0.001
	Intermediate	2126 (27.2)	411 (26.0)	1715 (27.5)	351 (27.6)	1364 (27.5)		
	High	3416 (43.7)	731 (46.3)	2685 (43.1)	473 (37.2)	2212 (44.7)		
	Unknown	1988 (25.5)	368 (23.3)	1620 (26.0)	400 (31.4)	1220 (24.6)		
**Lymphovascular Invasion**								
	Absent	2576 (33.0)	527 (33.4)	2049 (32.9)	545 (42.8)	1504 (30.4)	< 0.001	< 0.001
	Present	3045 (39.0)	480 (30.4)	2565 (41.2)	345 (27.1)	2220 (44.8)		
	Unknown	2187 (28.0)	573 (36.3)	1614 (25.9)	383 (30.1)	1231 (24.8)		
**Clinical Tumor Stage**								
	cT0	16 (0.2)	3 (0.2)	13 (0.2)	3 (0.2)	10 (0.2)	< 0.001	< 0.001
	cT1	865 (11.1)	138 (8.7)	727 (11.7)	232 (18.2)	495 (10.0)		
	cT2	2592 (33.2)	419 (26.5)	2173 (34.9)	598 (47.0)	1575 (31.8)		
	cT3	1362 (17.4)	224 (14.2)	1138 (18.3)	225 (17.7)	913 (18.4)		
	cT4	2520 (32.3)	663 (42.0)	1857 (29.8)	161 (12.6)	1696 (34.2)		
	Unknown	453 (5.8)	133 (8.4)	320 (5.1)	54 (4.2)	266 (5.4)		
**Clinical Nodal Stage**								
	cN0	1687 (21.6)	444 (28.1)	1243 (20.0)	530 (41.6)	713 (14.4)	< 0.001	< 0.001
	cN1	3314 (42.4)	578 (36.6)	2736 (43.9)	462 (36.3)	2274 (45.9)		
	cN2	985 (12.6)	171 (10.8)	814 (13.1)	86 (6.8)	728 (14.7)		
	cN3	1364 (17.5)	240 (15.2)	1124 (18.0)	142 (11.2)	982 (19.8)		
	Unknown	458 (5.9)	147 (9.3)	311 (5.0)	53 (4.2)	258 (5.2)		
**Tumor Subtype**								
	HR+/HER2-	3887 (49.8)	777 (49.2)	3110 (49.9)	602 (47.3)	2508 (50.6)	< 0.001	0.02
	HER2+	2403 (30.8)	479 (30.3)	1924 (30.9)	437 (34.3)	1487 (30.0)		
	TNBC	1238 (15.9)	237 (15.0)	1001 (16.1)	192 (15.1)	809 (16.3)		
	Unknown	280 (3.6)	87 (5.5)	193 (3.1)	42 (3.3)	151 (3.0)		
**Site of metastasis**								
	Bone only	3318 (42.5)	572 (36.2)	2746 (44.1)	583 (45.8)	2163 (43.7)	< 0.001	0.08
	Visceral or multiple sites	3216 (41.2)	859 (54.4)	2357 (37.8)	487 (38.3)	1870 (37.7)		
	Unknown	1274 (16.3)	149 (9.4)	1125 (18.1)	203 (15.9)	922 (18.6)		
**Sequence of systemic therapy**								
	Surgery first	2459 (31.5)	601 (38.0)	1858 (29.8)	385 (30.2)	1473 (29.7)	< 0.001	0.72
	Systemic therapy first	5349 (68.5)	979 (62.0)	4370 (70.2)	888 (69.8)	3482 (70.3)		
**Breast Surgery**								
	Partial Mastectomy	2250 (28.8)	769 (48.7)	1481 (23.8)	621 (48.8)	860 (17.4)	< 0.001	< 0.001
	Mastectomy	5558 (71.2)	811 (51.3)	4747 (76.2)	652 (51.2)	4095 (82.6)		
**Pathologic Tumor Stage**								
	pT0/ypT0	675 (8.6)	86 (5.4)	589 (9.5)	139 (10.9)	450 (9.1)	< 0.001	< 0.001
	pT1/ypT1	1120 (14.3)	187 (11.8)	933 (15.0)	246 (19.3)	687 (13.9)		
	pT2/ypT2	1785 (22.9)	299 (18.9)	1486 (23.9)	327 (25.7)	1159 (23.4)		
	pT3/ypT3	873 (11.2)	122 (7.7)	751 (12.1)	78 (6.1)	673 (13.6)		
	pT4/ypT4	1079 (13.8)	328 (20.8)	751 (12.1)	52 (4.1)	699 (14.1)		
	pTx	2270 (29.1)	556 (35.2)	1714 (27.5)	431 (33.9)	1283 (25.9)		
**Pathologic Nodal Stage**								
	pN0/ypN0			1635 (26.3)	642 (50.4)	993 (20.0)	-	< 0.001
	pN1/ypN1			1788 (28.7)	413 (32.4)	1375 (27.7)		
	pN2/ypN2			1331 (21.4)	81 (6.4)	1250 (25.2)		
	pN3/ypN3			989 (15.9)	20 (1.6)	969 (19.6)		
	pNx			485 (7.8)	117 (9.2)	368 (7.4)		
**Number of lymph nodes examined**				10.1 ± 8.0	3.5 ± 3.3	11.8 ± 8.1	-	< 0.001
**Number of lymph nodes positive**				5.0 ± 6.4	1.3 ± 2.3	5.9 ± 6.8	-	< 0.001
**Radiation therapy**								
	No	3219 (41.2)	828 (52.4)	2391 (38.4)	465 (36.5)	1926 (38.9)	< 0.001	0.04
	Yes	4221 (54.1)	677 (42.8)	3544 (56.9)	760 (59.7)	2784 (56.2)		
	Unknown	368 (4.7)	75 (4.7)	293 (4.7)	48 (3.8)	245 (4.9)		
**Chemotherapy**								
	No/Unknown	1120 (14.3)	305 (19.3)	815 (13.1)	181 (14.2)	634 (12.8)	< 0.001	0.14
	Yes	6688 (85.7)	1275 (80.7)	5413 (86.9)	1092 (85.8)	4321 (87.2)		
**Endocrine therapy**								
	No	3093 (39.6)	667 (42.2)	2426 (39.0)	493 (38.7)	1933 (39.0)	0.009	0.89
	Yes	4551 (58.3)	891 (56.4)	3660 (58.8)	753 (59.2)	2907 (58.7)		
	Unknown	164 (2.1)	22 (1.4)	142 (2.3)	27 (2.1)	115 (2.3)		

p1 - Comparison of no axillary surgery v. axillary surgery

p2 - Comparison between axillary surgery groups

Compared with no axillary surgery, patients undergoing axillary procedures were younger, less likely to be treated at community programs, and more likely to have ductal histology, lymphovascular invasion, bone-only metastases, lower clinical T stage, and to receive systemic therapy prior to surgery (70% vs. 62%, *p* < 0.001). Mastectomy was more common in the axillary surgery group (76% vs. 51%, *p* < 0.001). Sex and race distributions were similar between groups.

Among patients undergoing axillary surgery, those having ALND were older, more often non-White, and more likely to live in areas with higher educational attainment and median income (all *p* < 0.001). ALND was more frequent in patients with mixed/other histology, high-grade tumors, HER2 + subtype, and lymphovascular invasion (all *p* < 0.05). ALND patients also had higher clinical T stage and were less often cN0 (14% vs. 40%, *p* < 0.001).

### Nodal positivity

Of axillary surgery patients, 4,330/5,518 (78%) had positive lymph nodes, with a mean of 11.6 nodes examined and 6.5 positive (Table 3). Over half (56%) of node-negative patients had underwent ALND, highlighting potential overtreatment. Node positivity occurred in 58% of cN0 and > 80% of cN+ patients. Upfront surgery yielded higher nodal positivity than surgery after systemic therapy (87% vs. 74%, *p* < 0.001). HR+/HER2– tumors had the highest positivity rate (88%) compared with HER2+ (67%) and triple-negative (70%) (*p* < 0.001).

**Table 3:** Clinicopathologic characteristics of metastatic breast cancer patients who underwent surgery of the primary tumor and axilla stratified by whether positive nodes were found

		Overall	Pathologically positive nodes found		*p*
			No	Yes	
**n**		5,518	1,188	4,330	
**Age** (years)		54.4 ± 12.5	52.9 ± 12.6	54.9 ± 12.5	< 0.001
**Sex**					
	Male	114 (2.1)	19 (1.6)	95 (2.2)	0.2
	Female	5404 (97.9)	1169 (98.4)	4235 (97.8)	
**Race**					
	White	4226 (76.6)	921 (77.5)	3305 (76.3)	0.47
	Black	917 (16.6)	198 (16.7)	719 (16.6)	
	Asian	236 (4.3)	45 (3.8)	191 (4.4)	
	Other/Unknown	139 (2.5)	24 (2.0)	115 (2.7)	
**Charlson/Deyo Score**					
	0	4682 (84.8)	1029 (86.6)	3653 (84.4)	0.16
	1	653 (11.8)	125 (10.5)	528 (12.2)	
	2+	183 (3.3)	34 (2.9)	149 (3.4)	
**Insurance Status**					
	Not Insured	196 (3.6)	53 (4.5)	143 (3.3)	0.002
	Private Insurance	3068 (55.6)	695 (58.5)	2373 (54.8)	
	Medicaid	747 (13.5)	150 (12.6)	597 (13.8)	
	Medicare	1372 (24.9)	253 (21.3)	1119 (25.8)	
	Other Government	81 (1.5)	19 (1.6)	62 (1.4)	
	Unknown	54 (1.0)	18 (1.5)	36 (0.8)	
**No High School Degree**					
	>=17.6%	1110 (20.1)	220 (18.5)	890 (20.6)	0.3
	10.9% − 17.5%	747 (13.5)	284 (23.9)	1025 (23.7)	
	6.3% − 10.8%	1372 (24.9)	290 (24.4)	1005 (23.2)	
	< 6.3%	81 (1.5)	240 (20.2)	799 (18.5)	
	Unknown	250 (4.5)	154 (13.0)	611 (14.1)	
**Median Income**					
	< $40,227	950 (17.2)	189 (15.9)	761 (17.6)	0.28
	$40,227 - $50,353	1041 (18.9)	222 (18.7)	819 (18.9)	
	$50,354 - $63,332	1156 (20.9)	251 (21.1)	905 (20.9)	
	>=$63,333	1600 (29.0)	371 (31.2)	1229 (28.4)	
	Unknown	771 (14.0)	155 (13.0)	616 (14.2)	
**Institution Type**					
	Community Cancer Center	369 (6.7)	63 (5.3)	306 (7.1)	0.32
	Comprehensive Community Cancer Program	1952 (35.4)	417 (35.1)	1535 (35.5)	
	Academic/Research Program	1404 (25.4)	289 (24.3)	1115 (25.8)	
	Integrated Network Cancer Program	985 (17.9)	200 (16.8)	785 (18.1)	
**Histology**					
	Ductal	4873 (88.3)	1081 (91.0)	3792 (87.6)	< 0.001
	Lobular	425 (7.7)	43 (3.6)	382 (8.8)	
	Mixed/Other	220 (4.0)	64 (5.4)	156 (3.6)	
**Tumor Grade**					
	Low	183 (3.3)	26 (2.2)	157 (3.6)	< 0.001
	Intermediate	1549 (28.1)	261 (22.0)	1288 (29.7)	
	High	2372 (43.0)	531 (44.7)	1841 (42.5)	
	Unknown	1414 (25.6)	370 (31.1)	1044 (24.1)	
**Lymphovascular Invasion**					
	Absent	1831 (33.2)	593 (49.9)	1238 (28.6)	< 0.001
	Present	2404 (43.6)	237 (19.9)	2167 (50.0)	
	Unknown	1283 (23.3)	358 (30.1)	925 (21.4)	
**Clinical Tumor Stage**					
	cT0	12 (0.2)	4 (0.3)	8 (0.2)	0.02
	cT1	654 (11.9)	159 (13.4)	495 (11.4)	
	cT2	1948 (35.3)	440 (37.0)	1508 (34.8)	
	cT3	1022 (18.5)	210 (17.7)	812 (18.8)	
	cT4	1597 (28.9)	332 (27.9)	1265 (29.2)	
	Unknown	285 (5.2)	43 (3.6)	242 (5.6)	
**Clinical Nodal Stage**					
	cN0	1146 (20.8)	480 (40.4)	666 (15.4)	< 0.001
	cN1	2400 (43.5)	375 (31.6)	2025 (46.8)	
	cN2	714 (12.9)	95 (8.0)	619 (14.3)	
	cN3	976 (17.7)	190 (16.0)	786 (18.2)	
	Unknown	282 (5.1)	48 (4.0)	234 (5.4)	
**Tumor Subtype**					
	HR+/HER2-	2860 (51.8)	343 (28.9)	2517 (58.1)	< 0.001
	HER2+	1629 (29.5)	541 (45.5)	1088 (25.1)	
	TNBC	864 (15.7)	256 (21.5)	608 (14.0)	
	Unknown	165 (3.0)	48 (4.0)	117 (2.7)	
**Site of metastasis**					
	Bone only	2472 (44.8)	430 (36.2)	2042 (47.2)	< 0.001
	Visceral or multiple sites	2066 (37.4)	509 (42.8)	1557 (36.0)	
	Unknown	980 (17.8)	249 (21.0)	731 (16.9)	
**Sequence of systemic therapy**					
	Surgery first	1782 (32.3)	230 (19.4)	1552 (35.8)	< 0.001
	Systemic therapy first	3736 (67.7)	958 (80.6)	2778 (64.2)	
**Breast Surgery**					
	Partial Mastectomy	1315 (23.8)	378 (31.8)	937 (21.6)	< 0.001
	Mastectomy	4203 (76.2)	810 (68.2)	3393 (78.4)	
**Pathologic Tumor Stage**					
	pT0/ypT0	499 (9.0)	302 (25.4)	197 (4.5)	< 0.001
	pT1/ypT1	888 (16.1)	229 (19.3)	659 (15.2)	
	pT2/ypT2	1433 (26.0)	203 (17.1)	1230 (28.4)	
	pT3/ypT3	724 (13.1)	53 (4.5)	671 (15.5)	
	pT4/ypT4	709 (12.8)	62 (5.2)	647 (14.9)	
	pTx	1265 (22.9)	339 (28.5)	926 (21.4)	
**Axillary Surgery**					
	SLNB alone	1115 (20.2)	519 (43.7)	596 (13.8)	< 0.001
	ALND±SLNB	4403 (79.8)	669 (56.3)	3734 (86.2)	
**Number of lymph nodes examined**		10.5 ± 8.0	6.5 ± 6.0	11.6 ± 8.1	< 0.001
**Number of lymph nodes positive**		-	-	6.5 ± 6.6	-

In multivariable logistic regression, cN+ status was the strongest predictor of nodal positivity (OR > 7 for each nodal stage vs. cN0, Table 4). Other independent predictors included lobular histology, HR+/HER2– subtype, bone-only metastases, upfront surgery, mastectomy, and ALND. Similar predictors were found in a model limited to cN0 patients (data not shown).

**Table 4:** Multivariable logistic regression analyzing factors associated with finding positive axillary lymph nodes among metastatic breast cancer patients who underwent surgery of the primary tumor and axilla

		Entire cohort			cN0 cohort		
		OR	95% C.I.	*p*	OR	95% C.I.	*p*
**Age**		1.00	0.99–1.01	0.96	1.00	0.98–1.01	0.53
**Sex**							
	Male (reference)						
	Female	0.97	0.54–1.73	0.91	0.54	0.24–1.21	0.13
**Race**							
	White (reference)						
	Black	1.05	0.85–1.29	0.63	1.18	0.76–1.85	0.84
	Asian	1.09	0.74–1.60		1.13	0.49–2.65	
	Other/Unknown	1.38	0.82–2.35		0.81	0.33–1.97	
**Charlson/Deyo Score**							
	0 (reference)						
	1	1.11	0.88–1.41	0.58	0.78	0.52–1.17	0.23
	2+	1.16	0.74–1.82		1.51	0.72–3.17	
**No High School Degree**							
	>=17.6% (reference)						
	10.9% − 17.5%	0.87	0.69–1.10	0.48	0.94	0.60–1.46	0.29
	6.3% − 10.8%	0.95	0.75–1.20		0.90	0.58–1.42	
	< 6.3%	0.93	0.72–1.19		0.93	0.57–1.51	
**Institution Type**							
	Community Cancer Center (reference)						
	Comprehensive Community Cancer Program	0.99	0.71–1.38	0.61	0.77	0.42–1.43	0.48
	Academic/Research Program	1.05	0.74–1.48		0.86	0.45–1.64	
	Integrated Network Cancer Program	1.09	0.76–1.56		1.03	0.53–2.01	
**Histology**							
	Ductal (reference)						
	Lobular	1.69	1.16–2.45	0.003	1.95	1.16–3.29	< 0.001
	Mixed/Other	0.71	0.50–1.00		0.28	0.11–0.68	
**Tumor Grade**							
	Low (reference)						
	Intermediate	0.88	0.53–1.46	0.17	0.93	0.47–1.82	0.92
	High	0.74	0.45–1.22		0.89	0.45–1.75	
**Clinical Tumor Stage**							
	cT0 (reference)						
	cT1	1.62	0.43–6.12	0.11	1.69	0.21–13.78	0.95
	cT2	1.76	0.47–6.59		1.52	0.19–12.17	
	cT3	1.54	0.41–5.80		1.53	0.19–12.59	
	cT4	1.33	0.36–4.96		1.86	0.23–15.42	
**Clinical Nodal Stage**							
	cN0 (reference)						
	cN1	7.34	5.91–9.12	< 0.001			
	cN2	8.57	6.35–11.56				
	cN3	7.23	5.52–9.46				
**Tumor Subtype**							
	HR+/HER2- (reference)						
	HER2+	0.27	0.22–0.32	< 0.001	0.49	0.34–0.71	< 0.001
	TNBC	0.33	0.26–0.41		0.44	0.27–0.69	
**Site of metastasis**							
	Bone only (reference)						
	Visceral or multiple sites	0.79	0.66–0.94	< 0.001	0.72	0.53–0.98	0.05
**Breast Surgery**							
	Partial Mastectomy (reference)						
	Mastectomy	1.23	1.02–1.50	0.03	1.69	1.22–2.34	0.002
**Sequence of systemic therapy**							
	Surgery first (reference)						
	Systemic therapy first	3.36	2.74–4.11	< 0.001	3.70	2.62–5.22	< 0.001
**Axillary Surgery**							
	SLNB alone (reference)						
	ALND±SLNB	3.81	3.19–4.55	< 0.001	4.59	3.39–6.22	< 0.001

Notes: The multivariable analytic cohort includes N=4,204 patients, with 3,333 having any LN+ (79.3%)

This includes patients with non-missing data on cT, cN, and phenotype and also are age 40+ at diagnosis. (Patients age <40 years at dx do not have facility type recorded per NCDB PHI guidelines)

cN0 cohort includes N=926 patients, with 554 having any LN+ (59.8%)

### Survival analysis

Among patients surviving ≥ 12 months after diagnosis, median follow-up was 49.6 months from diagnosis (IQR, 32.9–74.5). Axillary surgery was associated with longer OS: median OS was 56.5 months for no axillary surgery versus 80.9 months for any axillary surgery (*p* < 0.001).

In multivariable Cox models, worse OS was independently associated with older age, Black race, higher comorbidity score, lower neighborhood income, lobular/other histology, advanced nodal stage, visceral or multiple metastatic sites, mastectomy, and systemic therapy prior to surgery (Table 5). Performance of any axillary surgery was independently associated with improved OS (HR 0.70; 95% CI 0.63–0.78; *p* < 0.001).

**Table 5:** Multivariable Cox proportional hazard model evaluating overall survival in patients who underwent surgery of the primary tumor

		Axillary Surgery (Yes vs No)			Axillary Surgery Type (None vs SLNB vs ALND±SLNB)		
		HR	95% C.I.	p	HR	95% C.I.	p
**Age**		1.01	1.00 - 1.01	0.002	1.01	1.00 - 1.01	0.002
**Sex**							
	Male (reference)						
	Female	1.11	0.83 - 1.46	0.49	1.10	0.83 - 1.46	0.51
**Race**							
	White (reference)						
	Black	1.22	1.09 - 1.36	0.001	1.21	1.08 - 1.35	0.002
	Asian	0.86	0.71 - 1.05		0.86	0.70 - 1.05	
	Other/Unknown	0.96	0.74 - 1.25		0.96	0.74 - 1.25	
**Charlson/Deyo Score**							
	0 (reference)						
	1	1.15	1.02 - 1.31	0.002	1.16	1.02 - 1.31	0.002
	2+	1.38	1.11 - 1.72		1.39	1.11 - 1.73	
**No High School Degree**							
	>=17.6% (reference)						
	10.9% - 17.5%	1.01	0.90 - 1.14	0.003	1.01	0.89 - 1.14	<0.001
	6.3% - 10.8%	1.01	0.87 - 1.16		1.01	0.87 - 1.16	
	< 6.3%	1.07	0.91 - 1.26		1.07	0.90 - 1.26	
**Median Income**							
	< $40,227						
	$40,227 - $50,353	0.88	0.78 - 1.01	<0.001	0.88	0.78 - 1.01	<0.001
	$50,354 - $63,332	0.85	0.74 - 0.98		0.85	0.74 - 0.98	
	>=$63,333	0.81	0.69 - 0.95		0.81	0.69 - 0.95	
**Institution Type**							
	Community Cancer Center (reference)						
	Comprehensive Community Cancer Program	1.01	0.90 - 1.14	0.003	0.95	0.82 - 1.11	0.008
	Academic/Research Program	1.01	0.87 - 1.16		0.80	0.68 - 0.95	
	Integrated Network Cancer Program	1.07	0.91 - 1.26		0.88	0.74 - 1.04	
**Histology**							
	Ductal (reference)						
	Lobular	1.50	1.30 - 1.73	<0.001	1.49	1.29 - 1.72	<0.001
	Mixed/Other	1.23	1.02 - 1.47		1.23	1.02 - 1.47	
**Tumor Grade**							
	Low (reference)						
	Intermediate	1.37	1.11 - 1.69	<0.001	1.49	1.29 - 1.72	<0.001
	High	1.90	1.55 - 2.33		1.23	1.02 - 1.47	
**Clinical Tumor Stage**							
	cT0 (reference)						
	cT1	1.28	0.42 - 3.91	0.06	1.26	0.41 - 3.81	0.08
	cT2	1.36	0.45 - 4.14		1.33	0.44 - 4.03	
	cT3	1.43	0.47 - 4.36		1.39	0.46 - 4.23	
	cT4	1.57	0.52 - 4.79		1.52	0.50 - 4.62	
**Clinical Nodal Stage**							
	cN0 (reference)						
	cN1	1.12	1.00 - 1.25	<0.001	1.08	0.97 - 1.21	0.04
	cN2	1.22	1.05 - 1.41		1.17	1.01 - 1.36	
	cN3	1.25	1.08 - 1.45		1.21	1.04 - 1.40	
**Tumor Subtype**							
	HR+/HER2- (reference)						
	HER2+	0.63	0.57 - 0.70	<0.001	0.63	0.57 - 0.70	<0.001
	TNBC	2.06	1.81 - 2.34		2.06	1.81 - 2.35	
**Site of metastasis**							
	Bone only (reference)						
	Visceral or multiple sites	1.26	1.15 - 1.37	<0.001	1.26	1.15 - 1.37	<0.001
**Breast Surgery**							
	Partial Mastectomy (reference)						
	Mastectomy	1.12	1.02 - 1.24	0.02	1.09	0.99 - 1.20	0.08
**Sequence of systemic therapy**							
	Before and after surgery (reference)						
	After surgery only	0.83	0.75 - 0.92	<0.001	0.84	0.76 - 0.93	<0.001
**Axillary Surgery**							
	No (reference)						
	Yes	0.70	0.63 - 0.78	<0.001			
	No (reference)						
	SLNB				0.60	0.51 - 0.69	<0.001
	ALND±SLNB				0.73	0.65 - 0.81	

Note: P-values for categorical variables reflect overall comparisons across categories rather than pairwise comparisons with the reference group. Hazard ratios (HR) represent the relative hazard of death; HR >1 indicates increased hazard of death and HR <1 indicates decreased hazard of death

Model 1 treats axillary surgery as a binary variable (yes vs. no); Model 2 models axillary surgery as a 3-level variable (none vs. SLNB vs.ALND±SLNB)

**Fig. 2:**
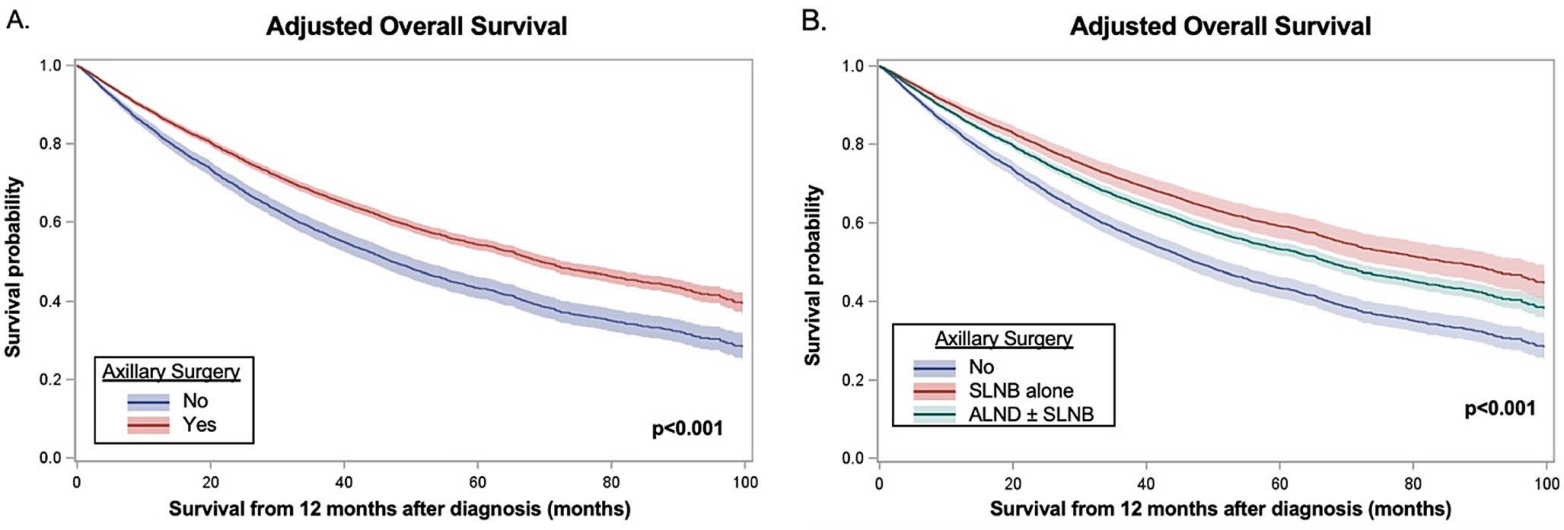
Overall survival among patients with de novo metastatic breast cancer undergoing surgery of the primary site and axilla. **A**: Adjusted Kaplan Meier curves comparing the axillary surgery and no axillary surgery groups; **B**: Adjusted Kaplan Meier survival curves comparing no axillary surgery, SLNB and ALND groups

Stratification by SLNB vs. ALND±SLNB produced similar findings. Survival curves from the multivariable models showed that in dnMBC patients undergoing primary site surgery, both SLNB and ALND were associated with improved OS compared with no axillary surgery (Fig. 2).

Propensity score–weighted analyses of OS using 9-, 12-, and 15-month landmark timepoints demonstrated consistent findings, with any axillary surgery associated with improved survival on binary analysis, and both SLNB and ALND associated with improved survival compared with no axillary surgery (Supplemental Table 2).

## Discussion

In this retrospective analysis of a large national database, we found that axillary surgery was commonly performed among patients with dnMBC who underwent primary tumor resection and was associated with longer OS compared with no axillary surgery. These findings must be interpreted with caution. Patients selected for primary site surgery represent a highly selected group with more favorable disease biology and overall prognosis. Moreover, even within this surgical cohort, patients undergoing axillary surgery differed meaningfully from those who did not, including younger age, lower comorbidity burden, lower tumor stage, and a higher likelihood of bone-only metastatic disease, These differences reflect important selection factors that are independently associated with improved survival and likely contribute to the observed association.

The optimal locoregional approach in dnMBC remains a subject of ongoing debate. Multiple retrospective series have suggested that resection of the primary tumor is associated with improved survival [5–7, 10, 16], with some reporting relative mortality risk reductions of 40–50% [10]. However, the survival benefit observed in these studies is inconsistently reproduced in randomized controlled trials [11–14]. The MF-07 trial, for example, initially demonstrated no difference in OS, but an apparent benefit emerged at 40 months and persisted at 10 years [12, 17]. Interpretation of this finding is limited by the absence of modern systemic therapy, short median OS in both arms, and possible imbalances in baseline characteristics. The recent E2108 trial, conducted in the contemporary therapeutic era, found no OS benefit for surgery of the primary tumor in dnMBC patients receiving optimal systemic therapy, and is widely considered the most robust evidence to date supporting the lack of OS benefit [11]. 

The role of axillary surgery in dnMBC has, unfortunately, been even less well studied. A 2020 meta-analysis of 16 studies (*n* = 16,692), which included both retrospective and prospective cohorts, reported no significant association between axillary surgery and OS (HR 0.82; 95% CI, 0.60–1.13), though there was substantial heterogeneity between the analyzed studies [15]. Our findings are consistent with prior retrospective studies showing improved survival in patients undergoing locoregional therapy, including axillary procedures [18], but the persistent gap between observational and randomized evidence underscores the strong likelihood that the observed survival differences in retrospective analyses reflect patient selection rather than a true therapeutic effect.

Our results reflect this pattern: patients undergoing axillary surgery were younger, had fewer comorbidities, lower tumor stage, and more frequently had bone-only metastases. While axillary surgery remained independently associated with improved OS after adjustment for measured factors, a direct causal effect is unlikely. In clinical practice, patients selected for locoregional surgery are typically those with more favorable disease biology, lower metastatic burden, and better response to systemic therapy. These factors are not fully captured in registry data and are strongly associated with improved survival. As a result, the observed association likely reflects residual confounding rather than a true therapeutic effect. Notably, the observed survival advantage persisted after propensity score weighting, suggesting that adjustment for measured confounders did not fully account for the association.

This interpretation is further supported by evidence from early-stage breast cancer, where randomized trials have consistently demonstrated that axillary surgery provides staging (which guides adjuvant therapies) and regional control but has not been shown to improve OS [19–22]. In the metastatic setting, where outcomes are driven by systemic disease burden and response to therapy, the biological plausibility of a survival benefit from axillary surgery is even more limited.

Axillary surgery, particularly ALND, is associated with well-documented morbidity. Reported rates of lymphedema after ALND range from 15% to over 25%, with higher risk when combined with nodal irradiation [23]. Additional complications include shoulder dysfunction, neuropathic pain, and chronic paresthesia, all of which can significantly impair quality of life [24, 25]. In de novo metastatic breast cancer, where systemic therapy selection is rarely altered based on axillary nodal status, the incremental oncologic value of axillary surgery is limited. In our cohort, most patients who underwent ALND did not first have sentinel lymph node biopsy (SLNB) performed, potentially exposing them to greater surgical morbidity, as some could have avoided ALND had SLNB been performed initially. Additionally, over half of patients who underwent axillary surgery had no pathologic nodal involvement, suggesting that many were exposed to surgical morbidity without clear oncologic benefit. As expected, clinically node-negative (cN0) patients were significantly less likely to have positive nodes than clinically node-positive patients, yet most cN0 patients underwent upfront ALND, highlighting the importance of careful clinical selection and opportunities to improve contemporary practice patterns. When axillary surgery is pursued, SLNB should be favored for patients without palpable adenopathy, as ALND in the absence of overt nodal disease confers added morbidity without proven oncologic gain. Overall, these findings suggest that routine axillary surgery in dnMBC, particularly upfront ALND, may represent surgical overtreatment in many patients, exposing them to morbidity without a clear impact on systemic management or survival.

Importantly, dnMBC alndrepresents a biologically heterogeneous disease, with substantial variation in tumor biology, metastatic burden, and response to systemic therapy. Prior studies suggest that select patients, such as those with limited metastatic disease, favorable tumor biology, or durable response to systemic therapy, may derive benefit from locoregional treatment of the primary tumor. In this context, axillary management should be individualized and considered within a multidisciplinary framework, balancing potential staging or local control benefits against the morbidity of axillary surgery. Our findings underscore the importance of careful patient selection and reinforce that axillary surgery should not be routinely applied to all patients with dnMBC undergoing breast surgery.

As systemic therapies continue to improve in efficacy and durability, the relative role of breast and axillary surgery in dnMBC may evolve. Integration of personalized systemic regimens with judicious surgical decision-making, ideally within the context of multidisciplinary discussion, will be essential. Future research should explore how modern systemic therapies modify the risk–benefit profile of axillary surgery and whether certain biologically defined subgroups could experience a true survival benefit.

Strengths of our study include the use of a large, contemporary national dataset, allowing robust subgroup analyses and multivariable adjustments not feasible in smaller cohorts. However, several limitations must be acknowledged. Importantly, selection bias persists not only in the decision to perform surgery but also in the choice of axillary management strategy. The NCDB lacks detailed information on metastatic disease burden, including the number and volume of metastatic lesions, as well as details regarding histologic confirmation and management of oligometastatic disease. While metastatic site was captured and incorporated into our analyses, this limited granularity restricts our ability to fully account for disease extent and may contribute to residual confounding. Clinical nodal staging in the NCDB lacks detail regarding imaging modality and physical examination findings, and misclassification of nodal status is possible. The retrospective design is subject to selection bias and confounding by indication, as well as time-related biases inherent to observational analyses. Although our landmark approach mirrors prior randomized trials, which enrolled patients with stable disease after initial systemic therapy, it results in a conditional survival analysis enriched for patients with more favorable disease biology and response to therapy. As such, guarantee-time bias may be introduced, and immortal time bias may not be fully eliminated despite this approach, potentially leading to overestimation of observed associations. Additional limitations relate to systemic therapy. Approximately one-third of patients in our cohort underwent surgery prior to receipt of systemic therapy, and the NCDB does not capture detailed information on systemic therapy regimens, including the use of contemporary targeted or immunotherapies. As a result, we are unable to account for variation in treatment selection or assess how advances in systemic therapy may influence these findings, and observed associations may reflect differences in treatment patterns rather than the effect of surgery itself. Taken together, these factors necessitate cautious interpretation of our findings, which are hypothesis-generating. Prospective randomized studies remain essential to define the role of axillary surgery in dnMBC.

## Conclusion

While our retrospective analysis suggests that patients with dnMBC who undergo both breast and axillary surgery were associated with improved overall survival compared to those receiving breast surgery alone, these findings should be interpreted with caution given the inherent limitations of observational data. Prospective studies and randomized controlled trials are needed to minimize selection bias and determine which subgroups may truly benefit from combined breast and axillary surgery. When axillary surgery is performed, sentinel lymph node biopsy should be the preferred approach in most patients without palpable adenopathy to reduce surgical morbidity. Multidisciplinary evaluation remains paramount to tailor surgical decisions to each patient’s clinical scenario and preferences. Ultimately, treatment strategies in dnMBC should not only aim to prolong survival, but also to preserve function, minimize treatment-related harm, and optimize quality of life.

## Supplementary Information

Below is the link to the electronic supplementary material.

Supplementary Material 1

## Data Availability

The data used in this study are derived from a de-identified National Cancer Database (NCDB) file and are not publicly available due to data-use restrictions and patient privacy protections but may be accessed by qualified investigators through application to the American College of Surgeons and the Commission on Cancer. The NCDB is a joint project of the Commission on Cancer of the American College of Surgeons and the American Cancer Society, both of which have not verified and are not responsible for the analytic methods or conclusions of the investigators.
