# Supplementary material for: Impact of axillary surgery on survival in de novo metastatic breast cancer with primary tumor resection
Source: Breast Cancer Res Treat. 2026 May 24;217(3):56. doi: 10.1007/s10549-026-07998-2 (PMC13287231; doi:10.1007/s10549-026-07998-2)
Supplement: Supplementary file 1 — Supplementary Material 1 [file 10549_2026_7998_MOESM1_ESM.docx]

Supplemental Table 1. Covariate comparison in the 12 month landmark cohort, prior to and after propensity score weighting.

|  |  | **Before PS weighting** | | **After PS weighting** | |
| --- | --- | --- | --- | --- | --- |
|  |  | *Axillary Surgery?* | | | |
|  |  | *No* | *Yes* | *No* | *Yes* |
| **Sex** | |  |  |  |  |
|  | Male | 2.1 | 2.4 | 2.5 | 2.3 |
|  | Female | 97.9 | 97.6 | 97.5 | 97.7 |
| **Race** | |  |  |  |  |
|  | White | 78.3 | 77.6 | 79.0 | 77.8 |
|  | Black | 15.8 | 16.1 | 14.7 | 16.0 |
|  | Asian | 3.7 | 4.0 | 4.2 | 4.0 |
|  | Other/Unknown | 2.2 | 2.2 | 2.1 | 2.2 |
| **Charlson Score** | |  |  |  |  |
|  | 0 | 86.6 | 84.2 | 82.1 | 84.5 |
|  | 1 | 10.3 | 12.4 | 14.3 | 12.1 |
|  | 2+ | 3.1 | 3.4 | 3.6 | 3.4 |
| **Education** | |  |  |  |  |
|  | Q1 highest% no HS diploma | 16.9 | 19.6 | 19.6 | 19.2 |
|  | Q2 | 23.8 | 23.4 | 24.6 | 23.5 |
|  | Q3 | 25.7 | 23.3 | 22.2 | 23.6 |
|  | Q4 lowest% no HS degree | 18.3 | 19.0 | 19.1 | 18.9 |
|  | Unknown | 15.2 | 14.6 | 14.5 | 14.8 |
| **Income** | |  |  |  |  |
|  | Q1 lowest median income | 14.9 | 17.0 | 16.6 | 16.6 |
|  | Q2 | 19.3 | 18.0 | 18.6 | 18.2 |
|  | Q3 | 20.1 | 21.5 | 21.0 | 21.2 |
|  | Q4 highest median income | 30.5 | 28.7 | 29.4 | 29.1 |
|  | Unknown | 15.2 | 14.8 | 14.5 | 14.9 |
| **Cancer Program Type** | |  |  |  |  |
|  | Community Cancer Center | 9.3 | 7.5 | 6.6 | 7.7 |
|  | Comprehensive Community Cancer Program | 46.9 | 41.0 | 42.6 | 42.3 |
|  | Academic/Research Program | 22.6 | 30.6 | 28.8 | 29.1 |
|  | Integrated Network Cancer Program | 21.1 | 21.0 | 22.0 | 21.0 |
| **Histology** | |  |  |  |  |
|  | Ductal | 87.6 | 88.1 | 88.1 | 88.0 |
|  | Lobular | 7.5 | 8.5 | 8.1 | 8.3 |
|  | Other | 5.0 | 3.4 | 3.7 | 3.7 |
| **Grade** | |  |  |  |  |
|  | 1 | 5.3 | 3.5 | 3.8 | 3.8 |
|  | 2 | 28.2 | 29.4 | 28.4 | 29.1 |
|  | 3 | 45.2 | 41.5 | 42.8 | 42.3 |
|  | Unknown | 21.3 | 25.5 | 25.0 | 24.8 |
| **Clinical T stage** | |  |  |  |  |
|  | cT1 | 9.5 | 13.3 | 12.8 | 12.6 |
|  | cT2 | 29.2 | 37.8 | 36.9 | 36.3 |
|  | cT3 | 15.8 | 18.9 | 18.2 | 18.4 |
|  | cT4 | 45.5 | 30.0 | 32.1 | 32.7 |
| **Clinical N stage** | |  |  |  |  |
|  | cN0 | 29.1 | 22.3 | 24.4 | 23.7 |
|  | cN1 | 41.7 | 46.1 | 46.3 | 45.2 |
|  | cN2 | 12.0 | 13.8 | 11.8 | 13.3 |
|  | cN3 | 17.2 | 17.8 | 17.5 | 17.7 |
| **Phenotype** | |  |  |  |  |
|  | HR+/HER2- | 52.0 | 55.9 | 53.0 | 54.9 |
|  | HER2+ | 34.7 | 29.9 | 30.9 | 30.9 |
|  | TNBC | 13.3 | 14.2 | 16.1 | 14.2 |
| **Lymphovascular Invasion** | |  |  |  |  |
|  | Not present | 35.3 | 34.1 | 36.1 | 34.5 |
|  | Present | 29.0 | 42.5 | 37.0 | 39.7 |
|  | Unknown | 35.6 | 23.4 | 26.9 | 25.8 |
| **Mets Site** | |  |  |  |  |
|  | Bone Mets Only | 40.5 | 45.7 | 43.0 | 44.7 |
|  | Visceral/Multiple/Other | 59.5 | 54.3 | 57.0 | 55.3 |
| **Surgery Type** | |  |  |  |  |
|  | Breast conserving surgery | 46.9 | 25.9 | 29.2 | 29.8 |
|  | Mastectomy | 53.1 | 74.1 | 70.8 | 70.2 |
| **First Treatment** | |  |  |  |  |
|  | Surgery First | 29.2 | 31.9 | 25.0 | 30.7 |
|  | Systemic First | 70.8 | 68.1 | 75.0 | 69.3 |

Supplemental Table 2. Survival analyses for overall survival and axillary surgery using landmark intervals of 12 mo after diagnosis as landmark starting point. (9 mo and 15 mo sensitivity analyses). Covariate adjustment using propensity scores which were applied using stabilized inverse probability weighting.

|  |  |  | **Model 1** | | | **Model 2** | | | | |
| --- | --- | --- | --- | --- | --- | --- | --- | --- | --- | --- |
|  |  |  | **Any Axillary Surgery (Yes vs No)** | | | **SLNB only vs no axillary surgery** | | **ALND±SLNB vs no axillary surgery** | | **p-value** |
|  | **N patients** | **N deaths** | **Hazard Ratio** | **95% CI** | **p-value** | **Hazard Ratio** | **95% CI** | **Hazard Ratio** | **95% CI** |  |
| 12 month landmark | 4870 | 1971 | 0.75 | 0.67-0.85 | <0.0001 | 0.67 | 0.53-0.83 | 0.77 | 0.67-0.87 | <0.0001 |
| 9 month landmark | 5061 | 2127 | 0.74 | 0.66-0.84 | <0.0001 | 0.68 | 0.55-0.84 | 0.75 | 0.67-0.85 | <0.0001 |
| 15 month landmark | 4669 | 1816 | 0.72 | 0.63-0.82 | <0.0001 | 0.61 | 0.48-0.78 | 0.74 | 0.65-0.84 | <0.0001 |

Propensity score covariates included sex, age at diagnosis, race, Charlson score, education (zip code based), income (zip code based), facility type, histology, grade, cT, cN, phenotype, LVI, metastatic site, primary surgery type, surgery-systemic therapy order.
